# Sexual Dimorphism in MAPK-Activated Protein Kinase-2 (MK2) Regulation of RANKL-Induced Osteoclastogenesis in Osteoclast Progenitor Subpopulations

**DOI:** 10.1371/journal.pone.0125387

**Published:** 2015-05-06

**Authors:** Bethany A. Herbert, Michael S. Valerio, Matthias Gaestel, Keith L. Kirkwood

**Affiliations:** 1 Department of Oral Health Sciences and the Center for Oral Health Research, Medical University of South Carolina, Charleston, South Carolina, United States of America; 2 Institute of Biochemistry, Hannover Medical School, Hannover, Germany; Los Angeles, UNITED STATES

## Abstract

Osteoclasts (OCs) are bone-resorptive cells critical for maintaining skeletal integrity through coupled bone turnover. OC differentiation and activation requires receptor activator of NF-kB ligand (RANKL) signaling through the p38 MAPK pathway. However the role of the p38 MAPK substrate, MAPK-activated protein kinase 2 (MK2), is not clearly delineated. Within the bone marrow exists a specific subpopulation of defined osteoclast progenitor cells (dOCPs) with surface expression of B220^-^Gr1^-^CD11b^lo/-^CD115^+ ^(dOCP^lo/-^). In this study, we isolated dOCPs from male and female mice to determine sex-specific effects of MK2 signaling in osteoclastogenesis (OCgen). Male *Mk2^-/-^* mice display an increase in the dOCP^lo^ cell population when compared to *Mk2^+/+^* mice, while female *Mk2^-/-^* and *Mk2^+/+^* mice exhibit no difference. Defined OCPs from male and female *Mk2^+/+^* and *Mk2^-/-^* bone marrow were treated with macrophage colony stimulation factor (M-CSF) and RANKL cytokines to promote OCgen. RANKL treatment of dOCP^lo^ cells stimulated p38 and MK2 phosphorylation. Tartrate-resistant acid phosphatase (TRAP) assays were used to quantify OC number, size, and TRAP enzyme activity post-RANKL stimulation. MK2 signaling was critical for male dOCP^lo^ OCgen, yet MK2 signaling regulated OCgen from female dOCP^-^ and CD11b^hi^ subpopulations as well. The functional gene, *Ctsk*, was attenuated in both male and female *Mk2^-/-^* dOCP^lo^-derived OCs. Conversely, MK2 signaling was only critical for gene expression of pre-OC fusion genes, *Oc-stamp* and*Tm7sf4*, in male OCgen. Therefore, these data suggest there is a sexual dimorphism in MK2 signaling of OCP subpopulations.

## Introduction

Osteoclasts (OCs) are multi-nucleated bone-resorbing cells that form by fusion of osteoclast progenitor cells (OCPs) arising from the hematopoietic cell lineage [1–3]. Under physiological conditions, mature OCs maintain bone turnover by eroding old bone so newer bone can be replaced by bone forming osteoblasts (known as coupled bone turnover) [4–6]. However in pathological conditions such as periodontal disease, osteoporosis, and arthritis, over-activation of OCs leads to excessive resorption and bone deterioration [7, 8].

Sexual dimorphisms exist in diseases associated with inflammation and bone loss. For example, post-menopausal women have decreased bone integrity and biomechanical strength than men [9]. Interestingly, men are at higher risk than women for periodontal disease, an oral disease characterized by inflammation and bone resorption [10]. One study provided evidence that estrogen replacement therapy is protective in women with periodontal disease [11]. OCs are the essential cell type involved directly in metabolic and pathological bone loss syndromes in both males and females.

It has long been thought that OCs mature from CD11b+ monocyte/macrophage bone marrow precursor cells [12]. More recently, the OCP isolated from murine bone marrow has been defined as B220^-^Gr1^-^CD11b^lo/-^CD115^+^ (dOCP^lo/-^) [13]. Macrophage colony stimulating factor (M-CSF) and receptor activator of NF-κB ligand (RANKL) cytokines drive survival, differentiation, and maturation of dOCP^lo/-^ cells *in vitro* [13]. At the cellular level, RANKL, produced by osteoblasts and stromal cells, induces intracellular signaling cascades by binding to its cognate receptor RANK on OCPs. Subsequently, RANKL stimulates osteoclastogenesis (OCgen) by activation of the intracellular mitogen activated protein kinase (MAPK) signaling pathway. All of the MAPKs including JNK, ERK, and p38 are required for OC differentiation under pathological and physiological conditions [14–17].

One of the three distinct MAPKs, specifically p38α/β, phosphorylates MAPK-activated protein kinase 2 (MK2). Both p38 MAPK and MK2 regulate inflammatory transcripts such as interleukin (IL-6), IL-1β, and tumor-necrosis factor (TNF)α by post-transcriptional modifications [18–20]. TNFα directly induces OCgen through p38 MAPK signaling and promotes RANKL gene expression in stromal cells [18, 21]. We previously reported that during inhibition of MK2, a major substrate of p38α/β MAPK, rats were protected against inflammation, OC formation, and bone loss during bacterial lipopolysaccharide (LPS) challenge [7]. Moreover, under normal physiological conditions, MK2 deficiency in male mice enhances trabecular bone and cortical thickness and decreases OCs and bone resorption when compared to WT control mice [22]. MK2 deficiency also protects female mice from trabecular bone loss in a murine ovariectomy estrogen-deficient model [22]. While MK2 signaling has mostly been studied under pathological conditions, its physiological function in sex differences are less known.

MK2 is activated by p38α/β MAPK phosphorylation through a wide variety of cell stimuli. In a stimulated cell, p38 MAPK phosphorylates and complexes with MK2 in the nucleus to activate MK2 and shuttle the complex back out into the cytoplasm [23, 24]. In the cytoplasmic subcellular compartment, MK2 phosphorylates target proteins involved in cell cycle control, proliferation, and differentiation. Nuclear factor of activated T-cells, cytoplasmic 1 (NFATc1), the “master transcription factor” of OCgen [25], binds to phospho (p)-p38 and co-localizes in the nucleus during RANKL/M-CSF-induced OCgen [26]. The p38/NFATc1 complex binds to the promoter of the Cathepsin K gene, *Ctsk* [26, 27]. Cathepsin K is an OC secreted proteolytic enzyme that promotes bone degradation. Although p38 MAPK regulates OCgen by direct interactions with NFATc1, more recent studies suggest that MK2 also plays a critical role in NFATc1 function during OCgen by regulating binding of NFATc1 to promoter regions of OC genes, *Acp5* (TRAP) and *Calcr* (calcitonin receptor) [22]. Other findings support that NFATc1 also binds to promoter regions on dendritic cell-specific transmembrane protein *Tm7sf4* (DC-STAMP), which is required for OC maturation into a multi-nucleated cell [28–30]. Both DC-STAMP and the more recently identified OC-STAMP are required for cell-cell fusion during OCgen [30, 31].

NFATc1 is among several transcription factors including PU.1, microphthalmia-associated transcription factor (MITF), NF-κB, and AP-1 (c-Fos/c-Jun) that regulate OC genes [2, 26, 27, 32–36]. M-CSF alone stimulates binding of PU.1 and MITF to the promoters of *Ctsk* and *Acp5* [27]. In addition to M-CSF, RANKL induces binding of p-MITF and p-p38 to promoter sites of *Ctsk*, preceding binding of NFATc1 to *Cstk* and *Acp5* promoters during OC differentiation [27]. Furthermore, α-tocopherol leads to p38α phosphorylation and MITF binding to the promoter of *Tm7sf4* in HEK293 cells [37]. Taken together, it is clear that MK2 and p38 MAPK are both critical regulators of OCgen, but the precise mechanism of this regulation during OCgen has previously been poorly defined.

Based on more recent findings that OCs mature from dOCPs with different osteoclastogenic potential, this study investigated the sexual dimorphism of MK2 signaling during OCgen from dOCPs. We demonstrated that MK2 positively regulated OCgen from the defined progenitor population, dOCP^lo^, from male and female mice by *Ctsk* gene expression. MK2 signaling was essential for expression of fusion genes, *Oc-stamp* and *Tm7sf4*, only in OCs derived from male mice.

## Materials and Methods

### Ethics Statement

Eight to twelve week old C57BL/6 *Mk2* ^+/+^ and *Mk2* ^*-/-*^ mice were obtained by material transfer from Germany [38], bred at the Medical University of South Carolina Animal Facility, and maintained in accordance with NIH guidelines. Animals were euthanized via CO_2_ asphyxiation followed by cervical dislocation. Mice were subject to food and tap water *ad libitum* and maintained under normal 12-hour light cycles. All experimental protocols were approved by the Institutional Animal Care and Use Committee (IACUC) at the Medical University of South Carolina under protocol number #2718.

### OCP Cell Sorting and Osteoclast Differentiation

Bone marrow cells were harvested from 8 to 12 wk old age and sex-matched *Mk2* ^+/+^ and *Mk2* ^-/-^ mouse tibia, femur, and humerus. Cells were plated at a density of 1 mouse / 10 cm tissue culture treated dish in alpha-MEM (Invitrogen, Carlsbad, CA, USA) containing 1% Penicillin/Streptomycin (P/S) and 10% HyClone Fetal Bovine Serum (FBS) (Fisher Scientific, Pittsburgh, PA, USA). HyClone FBS was used to avoid activation of OCPs by endotoxins contained in serum. Cells were incubated overnight in 5% CO_2_ at 37°C. Hematopoietic stem cells (HSCs) remaining in suspension were incubated with anti-CD11b-conjugated magnetic beads prior to sorting with the AutoMACS (Miltenyi Biotec Inc., San Diego, CA, USA). Cells were sorted by CD11b positive selection first into CD11b^hi^ and CD11b^lo/-^ populations using the Possel separation. The CD11b^lo/-^ population was sorted again by CD11b positive selection to separate dOCP^lo^ and dOCP^-^ cells using the slower Possel-s separation. Enriched dOCP populations were counted using a trypan blue dead cell exclusion assay and plated at a density of 1.5 x 10^6^/ cm^2^ and primed with 10 ng/mL recombinant mouse (rm) M-CSF (R&D Systems Inc., Minneapolis, MN, USA) for 2 days to form pre-OCs. Cells were differentiated into OCs with 25 ng/mL rmM-CSF and 50 ng/mL RANKL (R&D Systems Inc., Minneapolis, MN, USA) for up to 7 days. Cytokines were replaced every 2 days.

### Flow Cytometry

Mouse primary bone marrow cells were harvested from 8 to 12 week old male and female mice and plated overnight in alpha-MEM (Invitrogen, Carlsbad, CA, USA) containing 1% P/S and 10% HyClone FBS. Cells remaining in suspension (HSCs) were collected and stained with commercially available Miltenyi Biotec antibodies including anti-CD11b-APC (M1/70, rat IgG2b), anti-Gr-1/Ly6G-VioBlue (R86-8C5, rat IgG2b), anti-CD45R/B220-FITC (RA3-6B2, rat IgG2a), rat anti-mouse CD115 (Santa Cruz Biotec, Santa Cruz, CA, USA), and anti-rat Alexa Flour 488 (Invitrogen Molecular Probes, Eugene, OR, USA) following previously described methods [39].

### Western Blot and analysis

Defined OCP^lo^ cells were primed with M-CSF (10 ng/mL) for 2 days. Cell were serum starved in alpha-MEM containing 2% HyClone FBS and 0.1% P/S for 5 hours and then treated with RANKL (100 ng/mL). Cells were collected and lysed in RIPA buffer at 0, 10, 20, and 30 minutes post RANKL stimulation. Protein was quantified using the Pierce BCA Protein Assay Kit (Thermo Scientific, Rockford, IL, USA) and denatured at 95°C for 10 min in DTT and NuPage LDS Sample Buffer (Life Technologies, Grand Island, NY, USA). Twenty to thirty μg of protein was run on a 10% SDS-PAGE gel along with the Precision Plus Protein Dual Color standards (Bio-Rad Laboratories Inc., Hercules, CA, USA) and the Biotinylated Protein Ladder (Cell Signaling Technology Inc., Beverly, MA, USA). Gels were transferred to a nitrocellulose membrane and blocked in 5% skim milk for 1 hour at room temperature prior to incubating in primary antibody. Membranes were incubated in Cell Signaling primary antibodies: p-MK2 (Thr334, 27B7, rabbit mAb), p-p38 (Thr180/Tyr182, rabbit polyclonal), p38 (rabbit polyclonal), and GAPDH (14C10, rabbit mAb) at a 1:1000 dilution in 5% BSA in TBS-T overnight at 4°C. Blots were incubated in 1:000 anti-rabbit-IgG-HRP secondary antibody and anti-biotin-HRP to detect the biotinylated protein ladder in 5% skim milk at room temperature for 1 hour. Protein was visualized by chemiluminescence substrate (Thermo Scientific, Pittsburgh, PA, USA). Densiometric analysis was performed by capturing images using the Gel-Doc XR system and Quantity One 4.6.1 Software (Bio-Rad Laboratories Inc., Hercules, CA, USA).

### TRAP staining, enumeration, and activity

After sorting, dOCPs were seeded at a minimum of triplicate for OC differentiation in 96-well dishes. OCs were stained on days 3, 5, and 7 of differentiation with RANKL and M-CSF following the BD Biosciences protocol. OCs were washed and fixed with 10% glutaraldehyde for 15 min at 37°C. Cells were stained and 3 images were captured using QCapture Pro 7 (QImaging Corporation, Surrey, BC, Canada) at 100x magnification randomly per well. OCs were defined as TRAP positive with 3 or more nuclei. OCs were enumerated and measured using Adobe Photoshop CS5 (Adobe Systems Inc., San Jose, CA, USA). TRAP activity of dOCPs was measured using the Takara TRACP Kit following the manufacturer’s protocol (Takara Bioscience, Shiga, Japan). Cells were washed, processed, and activity was measured by absorbance at 405 nm using the VersaMax microplate reader and Softmax Pro 5 software (Molecular Devices Corporation, Sunnyvale, CA, USA)

### RNA isolation and Quantitative Polymerase Chain Reaction (qPCR)

Sorted dOCP^lo^ cells were plated at 1.5 × 10^6^ cells per tissue culture treated 35 mm dish. RNA was isolated from OCs differentiated for 3 days using TRIzol extraction methods (Invitrogen/Life Technologies, Carlsbad, CA, USA). RNA was quantified using NanoDrop and RT was performed using 300–1000 ng of RNA (Applied Biosystems Inc., Foster City, CA, USA) and diluted 3–10 times respectively. Samples were run with primers for *Acp5* (TRAP), *Ctsk* (Cathepsin K), *Tm7sf4* (DC-STAMP), *Oc-stamp*, *Nfatc1*, and *Tnfrsf11a* (RANK). RNA levels were normalized to house-keeping gene *Gapdh* using the StepOnePlus (Applied Biosystems Inc., Foster City, CA, USA). Relative mRNA levels were determined using the ΔΔCт values and represented as a fold change of control *Mk2* ^*+/+*^ cells treated with M-CSF only.

### Confocal microscopy and immunofluorescence

Sorted dOCP^lo^ cells were cultured and stained on 8-well chamber slides as previously described [39]. In brief, cells were primed with 10 ng/mL M-CSF for 2 days and treated with 25 ng/mL M-CSF or 25 ng/mL M-CSF and 50 ng/mL RANKL. Cells were fixed with 4% paraformaldehyde for 10 min at RT and permeablized with 0.5 M Triton X-100 and blocked with 5% normal goat serum. Cells were probed with primary antibodies: NFATc1 (7A6, mouse mAb, Santa Cruz Biotec, Santa Cruz, CA) and p-p38 (Thr180/Tyr182, rabbit polyclonal, Cell Signaling Technology Inc., Beverly, MA, USA). Fluorescence was detected using AlexaFluor-568 and AlexaFluor-488 secondary antibodies (Invitrogen Molecular Probes, Eugene, OR, USA). VECTASHIELD mounting media with DAPI (Vector Laboratories, Inc., Burlingame, CA, USA) was used to visualize nuclei. Images were captured using the Olympus FV10i laser scanning confocal microscope (Olympus Corporation, Center Valley, PA, USA). Three random images per well were captured for analysis. The Image J plug-in Jacop determined co-localization, where Pearson's correlation coefficient (r) values were analyzed between NFATc1, p-p38, and DAPI nuclear counterstain.

### Caspase 3/7 activity assay

Sorted dOCP^lo^ cells were plated in 96-well dishes and cultured for 5 to 7 days. Caspase 3/7 activity was measured using the Caspase-Glo 3/7 Assay following the manufacturer’s protocol for the 96-well white-walled plate (Promega Corporation, Madison, WI, USA). Treatment groups were blank-subtracted. Luminescence was detected using the Molecular Devices Spectramax and analyzed using Softmax Pro 5 software (Molecular Devices Corporation, Sunnyvale, CA, USA).

### Microcomputed Tomography

Right tibia were collected from male and female 3 or 6 month old *Mk2* ^*+/+*^ or *Mk2* ^*-/-*^ mice (n = 4). Tibia were fixed in 10% buffered formalin for 48 hours and stored in 70% ethanol. Tibia scanning and analysis was performed using the SCANCO Medical uCT 40. For the cortical bone, 104 slices with a resolution of 12 um were scanned starting 5 mm below the tibial growth plate at 55 kVp and 144 μA. The trabecular bone scan included a total of 209 slices starting 0.5 mm below the growth plate at 6 um resolution, 55 kVp, and 144 μA. Cortical scans were analyzed using midshaft analysis for bone volume faction (BV/TV) and cortical thickness (Th.) with a segmentation of 0.8/1 and threshold of 260. Trabecular scans were analyzed using bone morphometry analysis for trabecular parameters: number (Tb.N.), thickness (Tb.Th), connectivity density (Tb. Conn.D.), and bone volume fraction (Tb. BV/TV).

### Statistical Analysis

Data were analyzed with GraphPad Prism software, version 4.0. A two-tailed unpaired Student’s t-test was used to compare values between two groups. Four male and five female mice per treatment condition were used in RT-qPCR experiments and ΔCt values were used for statistical analysis. A one-tailed paired Student’s t-test was used to compare values between two groups for the TRAP enumeration. A one-way analysis of variance followed with a Bonferroni’s Multiple Comparison was used to compare multiple values. Results were expressed as mean ± SE or mean ± 95% confidence interval (CI) of at least three (n = 3) biological replicates for each experiment.

## Results

### MK2 regulates osteoclast size and number in defined osteoclast progenitor populations

**Fig 1:**
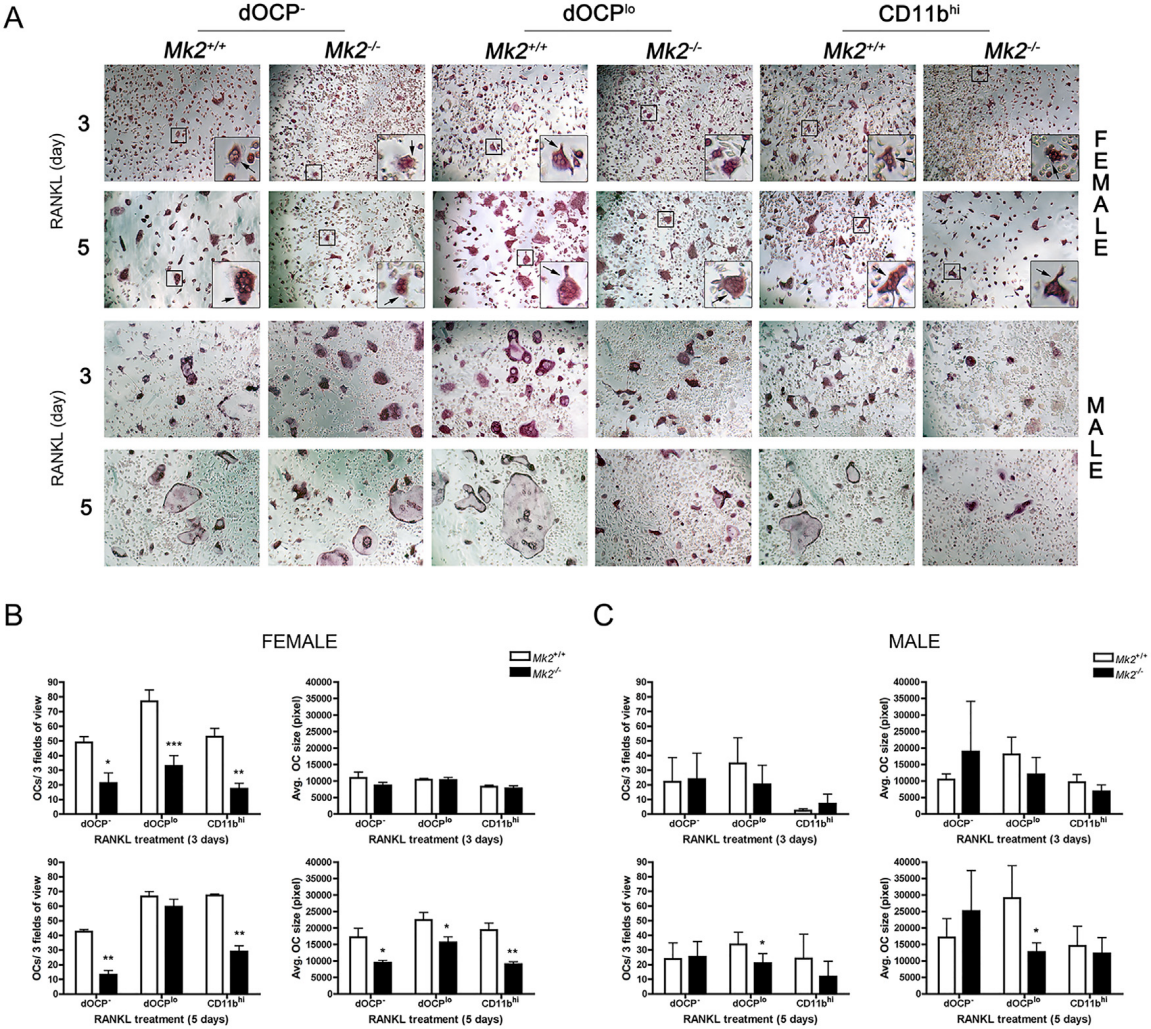
MK2 signaling differentially regulates osteoclast size and number from defined OCP cells. Cells were fixed, TRAP stained, and visualized by light microscopy. (A) Representative 100x images of female (top) and male (bottom) mouse bone marrow cells defined by CD11b surface expression driven to form osteoclasts (OCs) with M-CSF (25ng/mL) and RANKL (50 ng/mL). Female OCs (black arrow) were magnified 3x and inset (top). TRAP positive cells with three or more nuclei (OCs) from (B) female and (C) male mice were enumerated from three random images at days 3 (top) and 5 (bottom) of RANKL treatment. Osteoclast size from female (B, right) and male (C, right) was measured by pixels per osteoclast using Adobe PhotoShop CS. Data are expressed as means ± SE compared to *Mk2* ^*+/+*^ controls (*P≤0.05, **P≤0.01, ***P≤0.001).

To determine the role of MK2 signaling in physiological RANKL-induced OCgen from male and female *Mk2* ^*+/+*^ and *Mk2* ^*-/-*^ mice, we derived OCs from dOCP^-^, dOCP^lo^, and CD11b^hi^ cells. OC differentiation was visualized by TRAP staining (Fig 1A). M-CSF control treatments exhibited no TRAP staining (data not shown). OCs derived from female bone marrow cells were all decreased in number (*P*≤0.05), but not size in comparison to *Mk2* ^*-/-*^ OCs at day 3 of RANKL-induced differentiation (Fig 1A and 1B). By day 5 of RANKL treatment, female *Mk2* ^*-/-*^ OCs continued to develop from the *Mk2* ^*-/-*^ dOCP^lo^ population catching up to *Mk2* ^*+/+*^ dOCP^lo^ cell numbers (Fig 1A and 1B). MK2 deficiency led to a consistent decrease in formation of OCs derived from female dOCP^-^ and CD11b^hi^ cells on days 3 and 5 of RANKL treatment (*P*≤0.05, Fig 1A and 1B). The female OCPs continued to grow in size up to day 5, leading to a significant reduction in size in all *Mk2* ^*-/-*^ populations compared to respective *Mk2* ^*+/+*^ OCs. (Fig 1A and 1B). OCs derived from male *Mk2* ^*-/-*^ dOCP^lo^ cells were reduced in number and size compared to *Mk2* ^*+/+*^ OCs (*P*≤0.05) by day 5 of RANKL differentiation (Fig 1A and 1C). Both male and female *Mk2* ^*+/+*^ OCs increased in size, but not number, between days 3 and 5 of RANKL treatment. Although CD11b^hi^ and dOCP^-^ expressing cells were OCgenic, there was no significant difference in size and number between male *Mk2* ^*+/+*^ and *Mk2* ^*-/-*^ OCs (Fig 1A and 1C). Defined OCP^lo^-derived OCs were highest in number and size compared to CD11b^hi^ and dOCP^-^ derived OCs in both male and female assays. These data suggest that while MK2 signaling is most critical in the male dOCP^lo^ cells, all female OCP populations were regulated by MK2 signaling during OCgen.

### MK2 ablation is sex specific in regulation of dOCP lineage distribution

**Fig 2:**
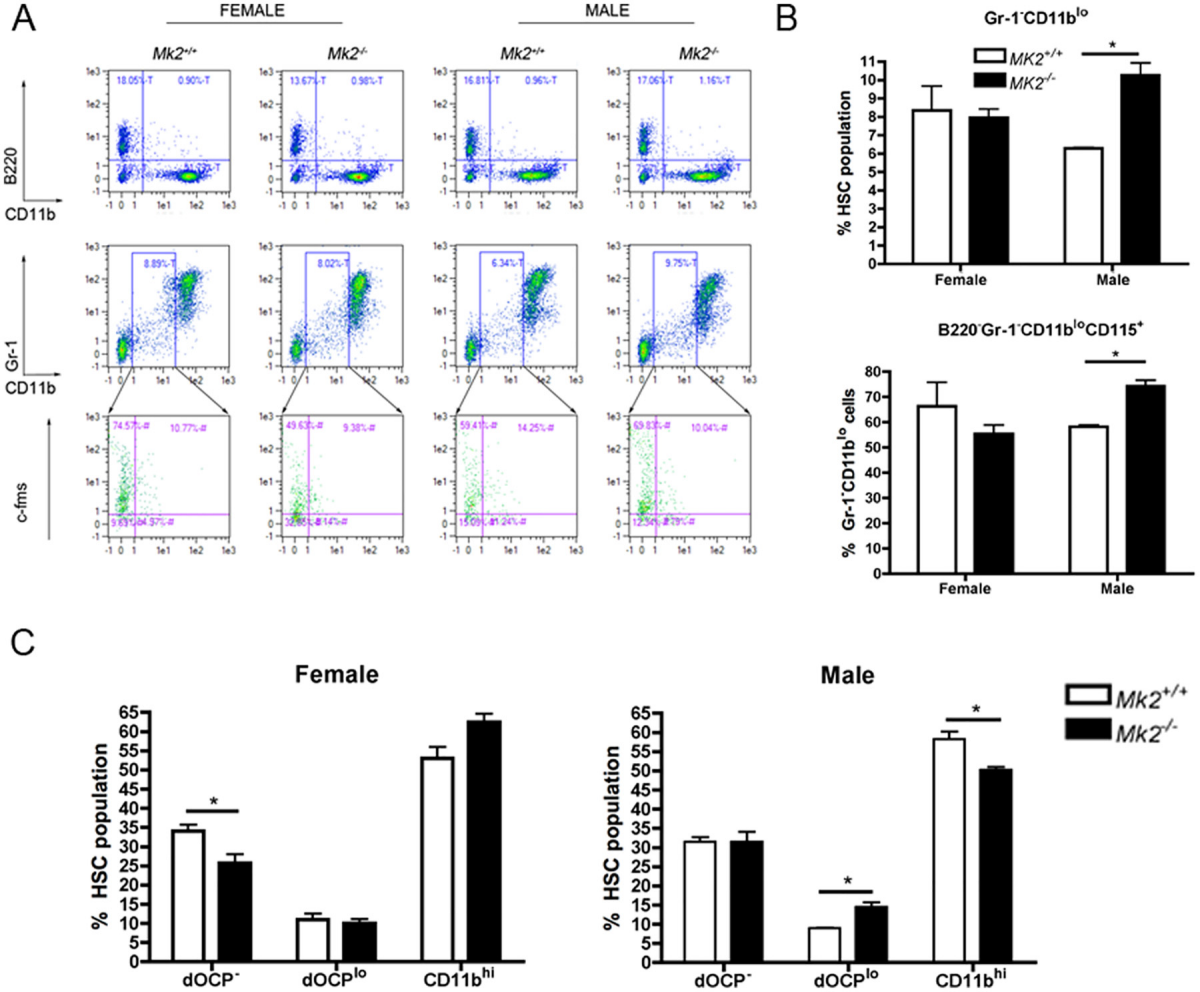
Ablation of MK2 increases dOCP^lo^ cells in male mice, but not female mice. (A) Representative flow cytometry of bone marrow from male and female *Mk2* ^*+/+*^ and *Mk2* ^*-/-*^ mice. (B) Enumeration of Gr-1^-^CD11b^lo^ cells as a percent of the hematopoietic stem cell population (HSC, top) and CD115+ as a percent of Gr-1^-^CD11b^*lo*^ cells (bottom). (C) Enumeration of CD11b surface expression in female (left) and male (right) mouse bone marrow cells. Data are expressed as means ± SE compared to *Mk2* ^*+/+*^ controls (*P≤0.05).

The mouse bone marrow-derived OCP population has previously been defined as B220^-^Gr1^-^CD11b^lo/-^CD115^+^ [13]. Flow cytometry showed that dOCP^lo^ cells, defined by CD11b^lo^ surface expression, were enriched for cell surface markers B220^-^Gr1^-^CD115^+^ (Fig 2A). We determined that *Mk2* ^*-/-*^ OC formation was not due to a basal deficiency dOCP^lo^ cells in male or female mice. Male *Mk2* ^*-/-*^ mice had a significant increase in dOCP^lo^ cells compared to male *Mk2* ^*+/+*^ mice (*P*≤0.05), but female mice showed no difference in this population (Fig 2B). Female *Mk2* ^*-/-*^ mice instead had a decrease in the dOCP^-^ lineage (*P*≤0.05), yet MK2 deficiency did not affect the dOCP^-^ population in male mice (Fig 2C). Instead male *Mk2* ^*-/-*^ mice had a deficient CD11b^hi^ bone marrow population compared to *Mk2* ^*+/+*^ mice (*P*≤0.05, Fig 2C). These data suggest that MK2 regulation of dOCP^lo^ cells is specific to male mice.

### RANKL induces phosphorylation of MK2 in dOCP^lo^ derived pre-OCs

**Fig 3:**
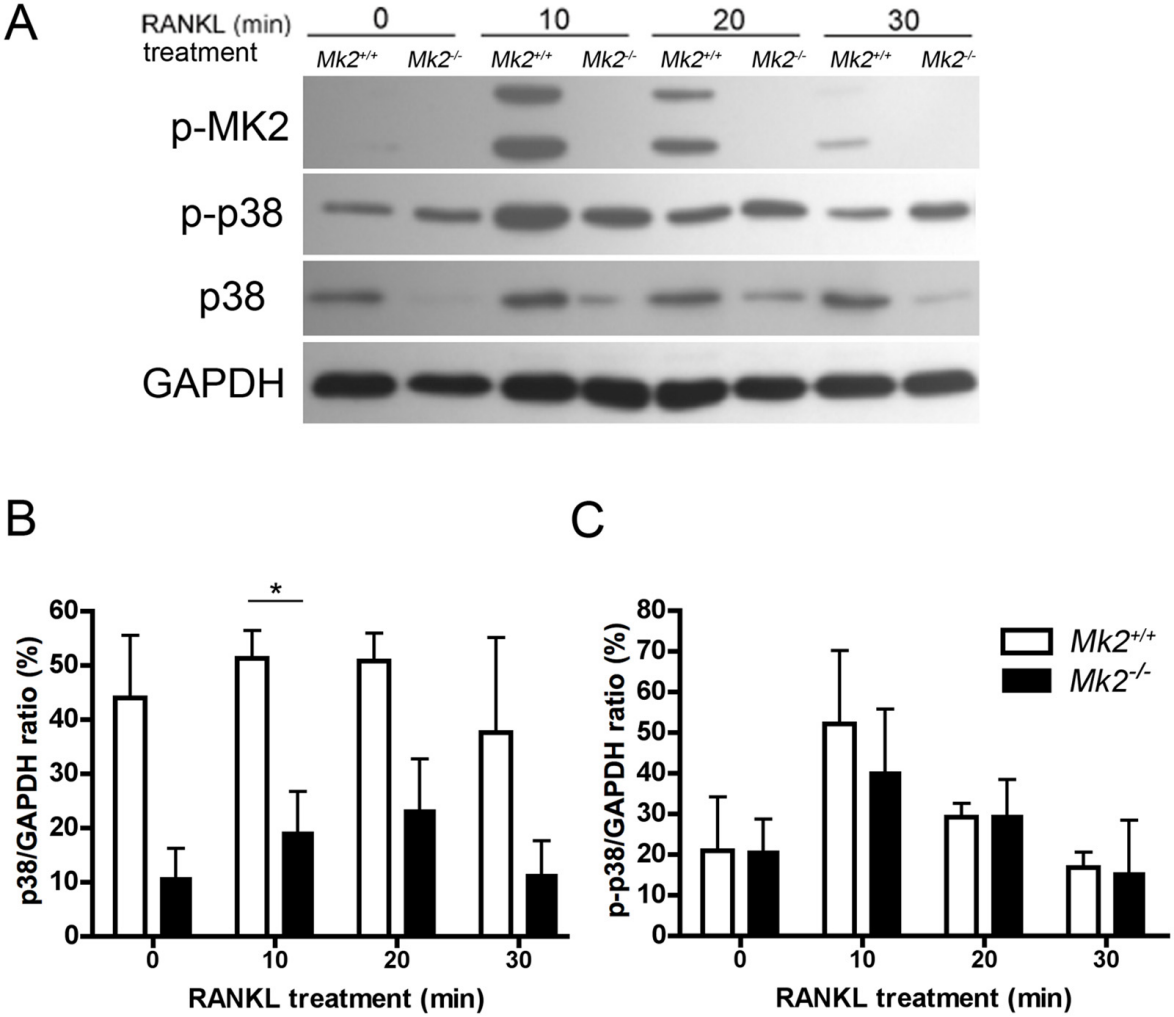
RANKL stimulates MK2 phosphorylation in dOCP^*lo*^ pre-osteoclasts. (A) Representative western blot of pre-osteoclasts from male mice primed with M-CSF (10 ng/mL) for two days and stimulated with RANKL (100 ng/mL) for the indicated minutes (min). Densiometric analysis of (B) p38 (left) and (C) p-p38 (right) as a percent of GAPDH. Data are expressed as means ± SE compared to *Mk2* ^*+/+*^ controls (*P≤0.05).

MK2 is necessary for p38 MAPK stabilization *in vivo* [40] and cellular localization between the nucleus into the cytoplasm *in vitro* [23]. To determine the effects of RANKL on p38 MAPK under MK2 deficiency, dOCP^lo^ cells from male and female *Mk2* ^*+/+*^ and *Mk2* ^*-/-*^ were driven to form pre-OCs. MK2 and p38 MAPK were rapidly phosphorylated in 10 minutes of RANKL treatment (Fig 3A and 3C, S2A and S2CFig). Interestingly, total p38 was significantly lower in *Mk2* ^*-/-*^ dOCP^lo^ cells compared to *Mk2* ^*+/+*^ cells (*P*≤0.05, Fig 3A and 3B, S2D Fig). Despite the lower amount of total p38 protein, p38 phosphorylation was not significantly different between dOCP^lo^ cells in the absence of MK2 (Fig 3C). MK2 and p38 were similarly phosphorylated in dOCP^lo^ cells derived from female mice (S2 Fig). These data demonstrate that MK2 is required for p38 MAPK stability during RANKL stimulation in dOCP^lo^ cells from male and female mice, but that p38 phosphorylation compensates the lower expression.

### RANKL induces NFATc1 nuclear localization

**Fig 4:**
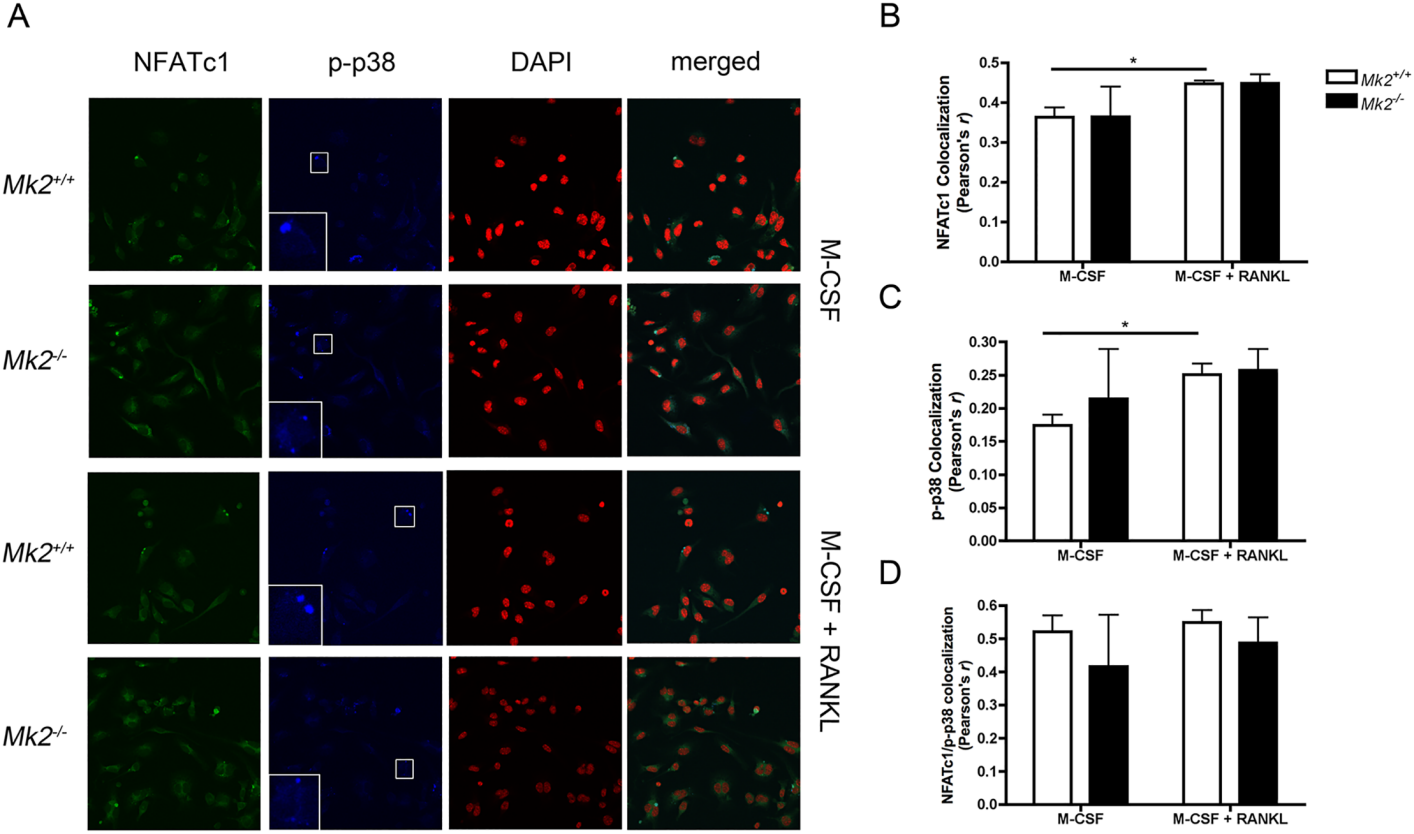
MK2 deficiency does not regulate NFATc1 colocalization. (A) Representative confocal images of male dOCP^*lo*^ cells treated with M-CSF (control) or M-CSF and RANKL for 30 minutes. A 3x magnified portion of p-p38 was inset (middle,left). (B) Pearson’s r correlation coefficients for NFATc1 and DAPI colocalization. (C) Pearson’s r correlation coefficients for p-p38 and DAPI colocalization. (D) Pearson’s r correlation coefficients for NFATc1 and p-p38 colocalization. Data are expressed as means ± SE compared to *Mk2* ^*+/+*^ controls (*P≤0.05).

Immunofluorescence of p-p38 and NFATc1 was performed to discern the effects of MK2 on cellular compartmentalization. RANKL treatment induced nuclear localization of NFATc1 and p-p38 in male *Mk2* ^*+/+*^ dOCP^lo^-derived pre-OCs. (Fig 4A, 4B, and 4C). MK2 deficiency did not regulate nuclear localization of NFATc1 or p-p38 as detected by similar Pearson’s correlation coefficient levels in *Mk2* ^*+/+*^ and *Mk2* ^*-/-*^ cells (Fig 4A, 4B, and 4C). MK2 signaling similarly did not regulate NFATc1 or p-p38 nuclear localization in female pre-OCs after RANKL and M-CSF treatment (data not shown). Phospho-p38 and NFATc1 colocalized with high correlation in the M-CSF control group and M-CSF and RANKL treatment group (Fig 4A and 4D).

### MK2 regulates expression of OC fusion genes

**Fig 5:**
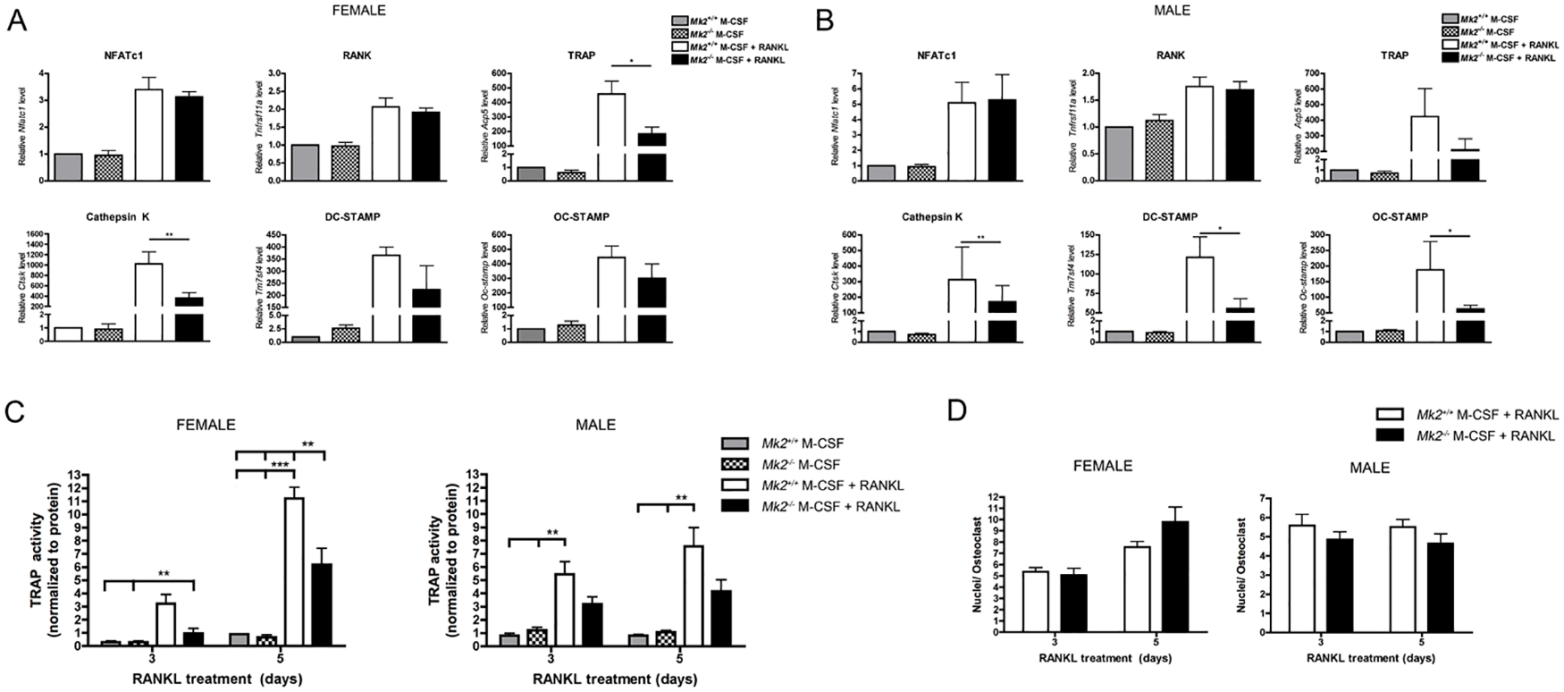
MK2 signaling regulates osteoclast fusion genes from male mice during osteoclast maturation. (A-D) Cells were treated as in Fig 1 for three days. Gene expression was determined by RT-qPCR in osteoclasts from (A) female and (B) male mice. (C) TRAP activity was assessed in female (left) and male (right) osteoclasts on day 3 and 5 post M-CSF and RANKL stimulation using the Takara TRACP Kit. (D) Nuclei were enumerated in female (left) and male (right) in osteoclasts, defined as TRAP positive cells with three or more nuclei. Data are expressed as means ± SE (*P≤0.05, **P≤0.01, ***P≤0.001).

Next, to demonstrate that MK2 regulates dOCP^lo^ fusion as well as OC formation, we performed RT-qPCR from mRNA isolated from dOCP^lo^-derived OCs from male and female mice after 3 days of RANKL stimulation. There was no detectable difference in male dOCP^lo^-derived OC size and number at this time-point during OCgen (Fig 1A and 1C); female OCs were fewer in number (*P*≤0.001) but displayed no difference in size (Fig 1A and 1B). Initial RT-qPCR results showed no difference in gene expression of *Nfatc1* and *Tnfrsf11a*, the receptor for RANKL in male and female-derived OCs (Fig 5A and 5B). TRAP activity, *Acp5*, and *Ctsk* were used as surrogate markers for OC function. Male *Mk2* ^*-/-*^ OCs had a trend toward decreasing gene expression of *Acp5*, and TRAP activity in the absence of MK2 (Fig 5B and 5C). Female *Mk2* ^*-/-*^ OCs were significantly reduced in both *Acp5* on day 3 (*P*≤0.05) and TRAP activity on days 3 and 5 during RANKL differentiation (*P*≤0.01, Fig 5A and 5C). While *Ctsk* gene expression was significantly attenuated in both male and female OCs (*P*≤0.01), MK2 deficiency led to an approximately 2.5 times decrease in *Mk2* ^*-/-*^ OCs from female mice compared to *Mk2* ^*+/+*^ OCs; a smaller change was 0.5 times less in *Mk2* ^*-/-*^ OCs from male mice compared to *Mk2* ^*+/+*^ OCs (Fig 5A and 5B). The magnitude of attenuated OC functional genes by MK2 deficiency was overall more profound in female mice than in male mice. Male *Mk2* ^*-/-*^ OCs also displayed over a 2-fold decrease in *Oc-stamp* and *Tm7sf4* gene expression (*P*≤0.05), which encode transmembrane proteins that promote mononuclear pre-OC fusion (Fig 5B). Interestingly, MK2 signaling in female OCgen did not regulate *Oc-stamp* and *Tm7sf4* gene expression (Fig 5A). We also found there was a trend toward decreasing nuclei per OC both 3 and 5 days post-RANKL stimulation in male OCs that was not evident in the female OCs (Fig 5D). The trend is consistent with the significant decrease in *Oc-stamp* and *Tm7sf*4 mRNA transcript levels in male OCgen, but not female OCgen. These results indicate that the overall *Mk2* ^*-/-*^ dOCP^lo^ population from male and female mice is less responsive to RANKL-induced OC formation when compared to *Mk2* ^*+/+*^ dOCP^lo^ cells. These results also support the notion that MK2 signaling regulates genes critical for OC function, but MK2 signaling preferentially regulates OC fusion gene expression during male dOCP^lo^ OCgen.

### Osteoclast formation decreases during differentiation

**Fig 6:**
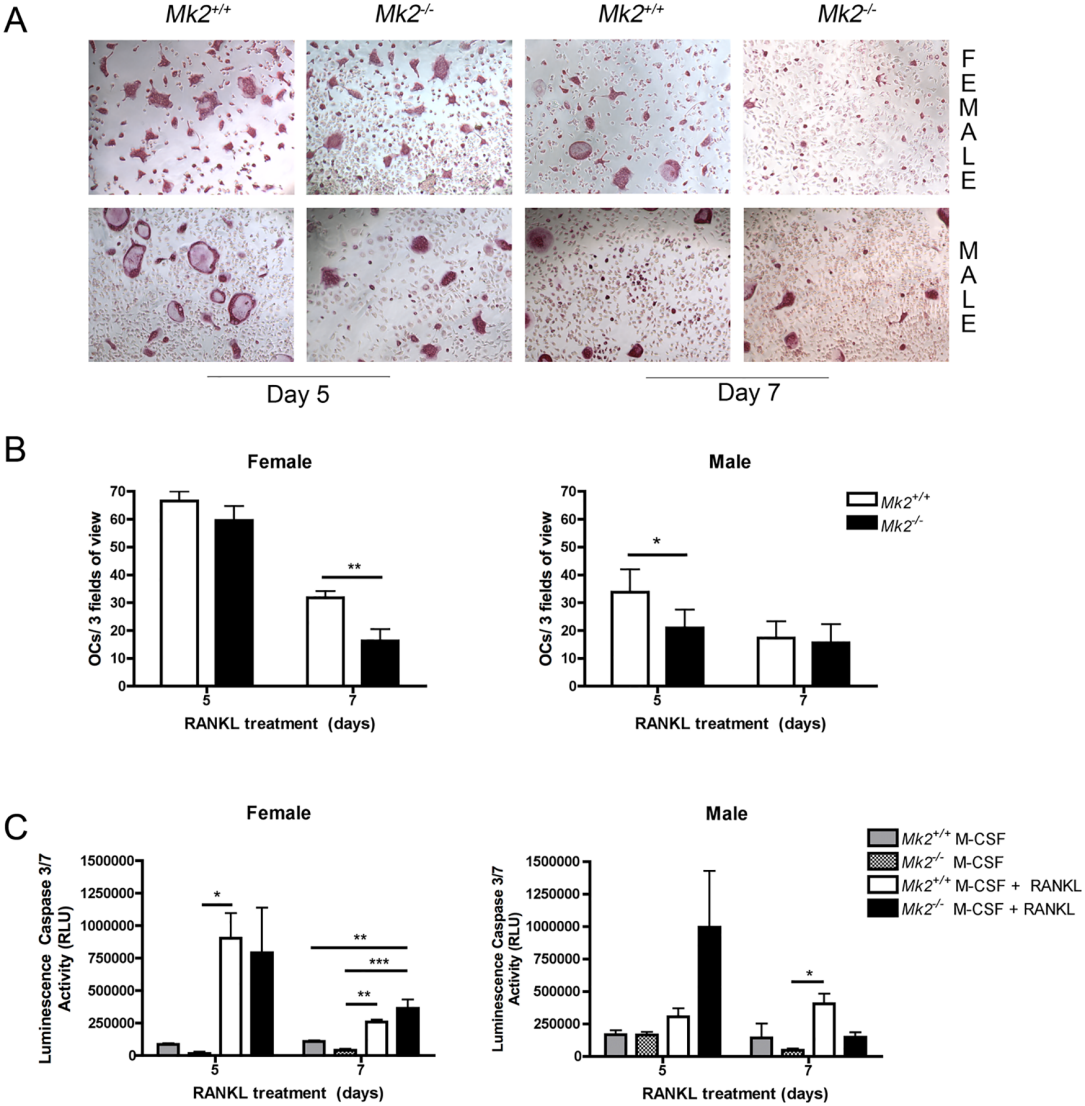
Osteoclast apoptosis during RANKL—induced differentiation. (A) Representative images of TRAP staining from female (top) and male (bottom) on days 5 and 7 of osteoclast culture (B) Osteoclast enumeration on days 5 and 7 from female (left) and male (right) dOCP^*lo*^ cells. (C) Caspase 3/7 activity on days 5 and 7 from female (left) and male (right) dOCP^*lo*^ cells. Data are expressed as means ± SE (*P≤0.05, **P≤0.01, ***P≤0.001).

To assess the role of MK2 signaling in OC apoptosis, dOCP^lo-^derived OCs were enumerated at days 5 and 7 of differentiation and apoptosis was detected by caspase 3/7 activity. Maximum OC numbers formed at day 3 of RANKL differentiation (Fig 1A, 1B, and 1C). At day 5 male *Mk2* ^*-/-*^ dOCP^lo^-derived OCs were fewer compared to *Mk2* ^*+/+*^ OCs (*P*≤0.05, Figs 1C, 6A and 6B), but female *Mk2* ^*-/-*^ *and Mk2* ^*+/+*^ OCs were the same number. By day 7 of differentiation, both male and female *Mk2* ^*-/-*^ and *Mk2* ^*+/+*^ OC numbers decreased (Fig 6B). Interestingly, female *Mk2* ^*-/-*^ OCs were significantly less than *Mk2* ^*+/+*^ OCs (*P*≤0.01, Fig 6B). RANKL increased caspase 3/7 activity in the female *Mk2* ^*+/+*^ OCs compared to the *Mk2* ^*-/-*^ M-CSF control group on day 5 of RANKL treatment (*P*≤0.05, Fig 6C). By day 7 of differentiation, the male *Mk2* ^*+/+*^ OCs had elevated caspase 3/7 activity compared to the *Mk2* ^*-/-*^ M-CSF control group (*P*≤0.05) and female *Mk2* ^*+/+*^ and *Mk2* ^*-/-*^ OCs had more caspase 3/7 activity compared to the *Mk2* ^*-/-*^ M-CSF control group (*P*≤0.01, Fig 6C). There were no significant differences at any time point in both genders for caspase 3/7 activity during RANKL-induced OCgen (Fig 6C). These data suggest that OC death has a sexual dimorphism, but MK2 signaling is not a key regulator.

## Discussion

This study demonstrates MK2 signaling differentially inhibits OC differentiation in mouse sexes. MK2 deficiency down-regulated male dOCP^lo^ OCgen, but not dOCP^-^ or CD11b^hi^ OCgen in cells from male mice. Female *Mk2* ^*-/-*^ OCs presented a different OCgen phenotype. OCs derived from male and female dOCP^lo^ cells formed the most OCs within 3 days when compared to *Mk2* ^*+/+*^ dOCP^-^ and dOCP^hi^, consistent with the report that dOCP^lo/-^ are the most OCgenic [41]. Female derived OCs were more numerous and smaller in size than male-derived OCs. Although the number and size of dOCP^lo^ OCs were not significantly different between male *Mk2* ^+/+^ and *Mk2* ^*-/-*^ cells at day 3 of RANKL stimulation, the male *Mk2* ^*-/-*^ OCs derived from dOCP^lo^ cells had less *Acp5*/TRAP and *Ctsk* mRNA transcripts at day 3, indicating that these *Mk2* ^*-/-*^ OCs have less activity. Similarly, female *Mk2* ^*+/+*^ OCs had more *Acp5*, *Ctsk*, and TRAP activity than the *Mk2* ^*-/-*^ OCs. The male *Mk2* ^*-/-*^ OCs were the only population that did not grow from days 3 to 5 indicating an insufficiency in male *Mk2* ^*-/-*^ dOCP^lo^-derived OCs to mature. While female *Mk2* ^*-/-*^ dOCP^lo^ OCs increased in size and number from days 3 to 5, the size remained significantly less than *Mk2* ^*+/+*^ OCs. OC numbers were fewer in OCs derived from female dOCP^-^ cells yet MK2-deficiency in male cells did not affect dOCP^-^ OCgen. Contrarily, MK2 deficiency in female dOCP^-^ cells led to a significant reduction in OC number at days 3 and 5 and size at day 5 of RANKL differentiation, further confirming the lineage and sex specific roles of MK2 signaling during OCgen.

One of the most interesting findings was that MK2 deficiency had differential regulation of CD11b surface expression of bone marrow cells from male and female mice. Male *Mk2* ^*-/-*^ mice had a significant increase in dOCP^lo^ cells compared to *Mk2* ^*+/+*^ male mice, whereas there were no differences in dOCP^lo^ cells from female mice. There are likely multiple factors contributing to the differential CD11b phenotype. Since MK2 deficiency is protective during estrogen-deficient bone loss in an ovariectomy mouse model [22], it is possible estrogen affects OCP development differently in female mice compared to males. MK2 maintains HSC quiescence [42], so an absence of MK2 could potentially lead to an increase in the dOCP^lo^ cells in the male mice that are derived from the hematopoietic niche. Despite the dOCP^lo^ gender discrepancy, male and female *Mk2* ^*-/-*^ mice maintain an enhanced skeletal phenotype compared to sex-matched *Mk2* ^*+/+*^ mice. Male *Mk2* ^*-/-*^ mice exhibit increased cortical and trabecular (Tb.) bone volume fraction (BV/TV) determined by microcomputed tomography results when compared to *Mk2* ^*+/+*^ mice whereas female mice show no significant difference (S1 Fig). Interestingly, adult HSC development is tightly regulated by the bone marrow niche cellular components and size [43]. Reduced femur cavity size in *Bmpr1a* mutant mice led to an overall reduction in HSCs [43]. Sexual differences in niche size as indicated by increased trabecular number (Tb.N.) in male and female *Mk2* ^*-/-*^ mice (S1 Fig) may also be contributing to a decrease in female dOCP^-^ expressing cells or male CD11b^hi^ cells, that are derived from the hematopoietic lineage. Although the *Mk2* ^*-/-*^ male mice have an enhanced percentage of dOCP^lo^ cells *in vivo*, these cells are indeed not contributing to increased OC formation and subsequent bone loss indicated by the increased trabecular and cortical bone in *Mk2* ^*-/-*^ males.

We next aimed to look at RANKL-induced phosphorylation of MK2 in the dOCP^lo^ population. MK2 was quickly phosphorylated by RANKL, but upon RANKL stimulation of dOCP^lo^ cells, total p38 MAPK remained at lower expression levels in *Mk2* ^*-/-*^ cells. Phoshpo-p38 levels however did not change. Both *Mk2* ^+/+^ and *Mk2* ^*-/-*^ cells had phosphorylation of p38 by 10 minutes of RANKL stimulation, showing no differences in male and female phosphorylation kinetics. These data suggest that although total p38 levels are reduced during MK2 deficiency due to the role of MK2 in stabilizing p38, there is still enough total protein present for normal phosphorylation of p38 activated by RANKL. The antibody used to detect total p38 MAPK targets p38α, -β, and –γ, but not -δ isoforms. MK2 deficient tissues and cells have considerable reduction in p38α levels, which has a stable intracellular interaction with MK2 [23, 40, 44, 45]. The four isoforms of p38 are not equally activated and expressed in all tissue types. For example, in tissues from rheumatoid arthritis patients, macrophages express p38α and— γ, yet fibroblasts have p38β and- γ isoforms [46]. The p38α MAPK is the main isoform detected in OC precursor cells primed with M-CSF and inhibition of p38α abrogates M-CSF/RANKL-induced OCgen [17, 47]. We also often see that p38α phosphorylation compensates the decreased p38α level in MK2/3 knockout mice [48]. This could be simply due to an increased fraction of phosphorylated p38α in the MK2 knockout mice compared to wild-type [48]. Therefore, it is likely the lower levels of p38 detected in *Mk2* ^*-/-*^ pre-OCs is caused by destabilization of the p38α isoform. Since p38 γ is also expressed in macrophages, which can be derived from the same lineage as pre-OCs, it is possible that p38 isoforms compensate for the destabilization of others that allow for normal phosphorylation of p38 in the *Mk2* ^*-/-*^ cells.

During MK2 deficiency, p38 MAPK was deregulated and p-p38 levels were normal, so we next aimed to determine whether p-p38 subcellular compartmentalization was disrupted because MK2 regulates p38 shuttling between the cytoplasm and nucleus [23]. NFATc1 along with p-p38 co-localize to regulate OC gene transcription [17, 27]. NFATc1 is directly phosphorylated by p38 and binds to p38 and PU.1 [26]. Another MAPK regulator, a MAPK phosphatase MKP-1, promotes NFATc1 nuclear localization in sorted dOCPs [39]. Upon investigating the role of MK2 signaling in NFATc1 nuclear localization we found that MK2 does not regulate early NFATc1 nuclear localization in male or female dOCP^lo^ cells, indicating MK2 signaling does not disrupt NFATc1/p-p38 shuttling during OCgen and the early activation of the p38/MK2 MAPK axis is similar in male and female cells.

This study reports that MK2 signaling regulates mRNA expression of both OC fusion genes, *Oc-stamp* and *Tm7sf4* during OCgen in male dOCP^lo^ cells. MK2 deficiency also led to a reduction in *Ctsk*, a transcript critical for OC function. TRAP activity was decreased in the male and female *Mk2* ^*-/-*^ dOCP^lo^ cells indicating these OCs were less functional. The TRAP activity assay was utilized as a surrogate functional assay for OCs and is correlated with OCgen bone resorption pit assays *in vitro* [39]. TRAP is an essential enzyme for OC function, which has been demonstrated in the *Acp5* (TRAP)-null mouse. *Acp5* knockout mice have an osteopetrotic skeletal phenotype marked by a lack of functional OCs [49]. TRAP activity levels in *Mk2* ^*-/-*^ female mice were similar to the activity of the male *Mk2* ^*+/+*^ mice. The OC activity may correlate with differing bone phenotypes between male and female tibia (S1 Fig). Indeed, the *Mk2* ^*-/-*^ female mice have a similar bone phenotype to the *Mk2* ^*+/+*^ male mice (S1 Fig). Although MK2 regulates *Tnfrsf11a* gene expression [22], there was no difference detected in the dOCP^lo^ population in male or female mice, suggesting MK2 may differentially regulate OC-genes, such as *Tnfrsf11a* in the other dOCP lineages. MK2 absence did not entirely abrogate OCgen suggesting there is enough of the transcript present to promote incomplete OCgen in the *Mk2* ^*-/-*^ cells. Fusion proteins OC-STAMP and DC-STAMP are required for mononuclear cells to fuse into a multi-nucleated OC. The trend toward a decrease in nuclei per male *Mk2* ^*-/-*^ OC compared to *Mk2* ^*+/+*^ is correlated with a decrease in *Oc-Stamp* and *Tm7sf4* (DC-STAMP) that will hinder cell fusion during OCgen. Based on the finding that MK2 regulates binding of NFATc1 to promoter regions of *Calcr* and *Acp5* [22] we reasoned MK2 may also regulate NFATc1 binding to the promoters in male dOCP^lo^ cells including *Ctsk*, *Oc-stamp*, and *Tm7sf4*, which could explain their down-regulation during MK2 deficiency. In female dOCP^lo^ cells, *Oc-stamp* and *Tm7sf4* were not regulated by MK2 signaling, but *Acp5* and *Ctsk* were highly affected. It is also possible MK2 regulates other proteins in the OC transcription complexes, including c-Fos whose binding to the *Calcr* and *Acp5* promoter regions is also regulated by MK2 [22]. *Ctsk* and *Dc-stamp* also contain an AP-1 binding domain, on which c-Fos and c-Jun hetero- or homodimers may bind [29, 36].

Lastly, to determine OC cell death, OCs were enumerated and caspase 3/7 activity was detected as a marker for OC apoptosis. Since OC formation continues until day 5, we measured caspase 3/7 activity on days 5 and 7 after RANKL stimulation. At day 5 there was a significant reduction in male *Mk2* ^*-/-*^ OCs but not female. Unlike male OCs, female OCs had no difference in caspase 3/7 activity on day 5. There was an increasing trend in caspase 3/7 in male *Mk2* ^*-/-*^ OCs at day 5 suggesting that apoptosis may be contributing to differences seen at this day when OCgen is teetering on OC formation and dying. By day 7, both *Mk2* ^*+/+*^ and *Mk2* ^*-/-*^ OCs began to decrease in number with female *Mk2* ^*-/-*^ OCs declining more than *Mk2* ^*+/+*^ OCs. Mononuclear cells within the enriched OC culture could also have contributed to the caspase 3/7 levels, but since cells were initially seeded at the same density, any contributing caspase 3/7 activities in the cultures should be equal among all populations and treatment groups. These results suggest not only do male and female mice have sexual dimorphism during OCgen, but they also differ in survival irrespective of MK2.

We demonstrated significant novelty in MK2 signaling during OCgen in addition to previous findings of Braun et al. (2013). Previous studies identified MK2 as a key regulator of OCgen using whole bone marrow populations from male mice [22]. This study delineated OCP populations from male and female mice and detected a sexual dimorphism. MK2 signaling regulated male dOCP^lo^ OCgen and female dOCP^-^, dOCP^lo^, and CD11b^hi^ OCgen at different time-points during RANKL-induced differentiation. It is essential to discern differences between males and females because bone loss diseases such as periodontal disease and osteoporosis exhibit gender biased. Male and females also have different physiological skeletal phenotypes. We identified novel gene transcripts *Tm7sf4* and *Oc-stamp* that were regulated by MK2 in the male dOCP^lo^ population but not in the female dOCP^lo^ population. It was previously described that MK2 regulates binding of NFATc1 and c-Fos to the promoters of *Calcr* and *Acp5* [22]. The cellular compartmentalization of p38 MAPK and NFATc1 are absolutely essential for function, and our results prove MK2 signaling does not regulate nuclear localization of NFATc1 indicating that NFATc1 promoter binding is not affected by its lack of ability to enter the nucleus during OCgen. We also found MK2 signaling has a larger role in OCgen than OC apoptosis.

In conclusion, MK2 signaling is critical for OCgen from the male dOCP^lo^ cells and regulates OC fusion genes *Oc-stamp* and *Tm7sf4*. Moreover, therapeutic agents are currently being designed to target MK2 in inflammatory diseases [50]. Drug therapy for skeletal diseases is limited due to deleterious side effects of inhibiting bone turnover, thus increasing the potential of pathological fractures due to reduced skeletal remodeling. These studies provide a better understanding of the mechanisms of the sexual dimorphism of MK2 signaling during physiological RANKL-induced OCgen and delineate sex differences with the potential for targeting MK2 in gender bias OC-driven diseases.

## Supporting Information

S1 Fig MK2 signaling regulates skeletal morphology. (A) Representative microcomputed tomography 3-D reconstruction from 3 and 6 month old male and female trabecular bone. (B) Trabecular number (Tb.N), trabecular thickness (Tb.th.), trabecular bone volume fraction (Tb. BV/TV) and trabecular connectivity density (Tb. Conn.D.) were quantified using Scanco Medical Software. (C) Representative microcomputed tomography 3-D reconstruction of cortical bone from 3 and 6 month old male and female mice. (D) Cortical bone volume fraction (BV/TV) and cortical thickness (Th.) were measured using Scanco Medical Software. Data are expressed as means ± SE compared to *Mk2* ^*+/+*^ controls (*P≤0.05, **P≤0.01, ***P≤0.001)
(TIF)

S2 Fig RANKL stimulates MK2 phosphorylation in dOCP^lo^ pre-osteoclasts. (A-D) Whole images of representative western blots showing the molecular weight ladder. (A) p-MK2 was detected only in *Mk2* ^*+/+*^ samples near 49 kDa of the biotinylated ladder. The two bands detected may be different phosphorylated forms of MK2. (B) p-p38 was detected at 43 kDa of the biotinylated ladder. (C) GAPDH was used as the loading control and detected at 37 kDA using the color ladder and transferred to the film (left) or biotinylated ladder (right). (E) Total p38 was detected at 37 kDa using the biotinylated ladder.
(TIF)
