# Supplementary figures and images for: Sexual Dimorphism in MAPK-Activated Protein Kinase-2 (MK2) Regulation of RANKL-Induced Osteoclastogenesis in Osteoclast Progenitor Subpopulations
Source: PLoS One. 2015 May 6;10(5):e0125387. doi: 10.1371/journal.pone.0125387 (PMC4422514; doi:10.1371/journal.pone.0125387)

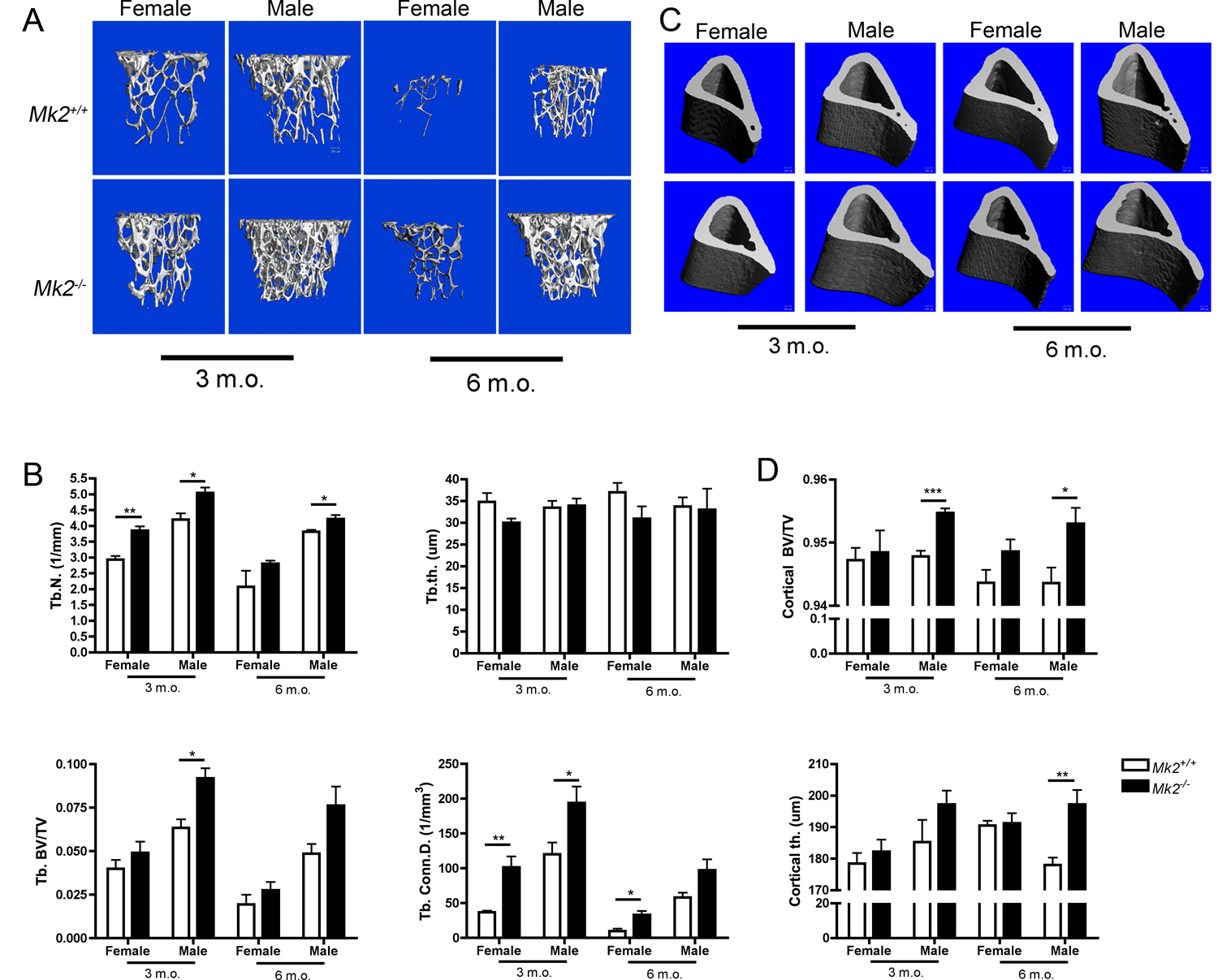

Supplement: S1 Fig — (A) Representative microcomputed tomography 3-D reconstruction from 3 and 6 month old male and female trabecular bone. (B) Trabecular number (Tb.N), trabecular thickness (Tb.th.), trabecular bone volume fraction (Tb. BV/TV) and trabecular connectivity density (Tb. Conn.D.) were quantified using Scanco Medical Software. (C) Representative microcomputed tomography 3-D reconstruction of cortical bone from 3 and 6 month old male and female mice. (D) Cortical bone volume fraction (BV/TV) and cortical thickness (Th.) were measured using Scanco Medical Software. Data are expressed as means ± SE compared to Mk2 +/+ controls (*P≤0.05, **P≤0.01, ***P≤0.001) (TIF) [file pone.0125387.s001.tif]

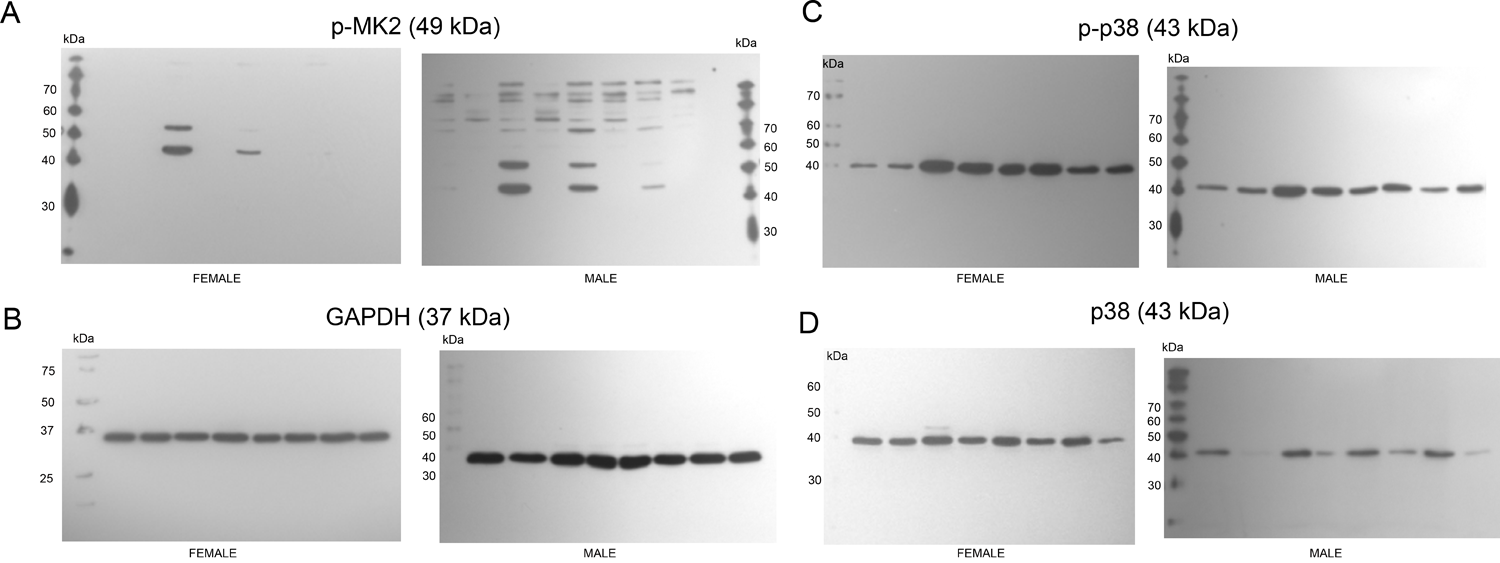

Supplement: S2 Fig — (A-D) Whole images of representative western blots showing the molecular weight ladder. (A) p-MK2 was detected only in Mk2 +/+ samples near 49 kDa of the biotinylated ladder. The two bands detected may be different phosphorylated forms of MK2. (B) p-p38 was detected at 43 kDa of the biotinylated ladder. (C) GAPDH was used as the loading control and detected at 37 kDA using the color ladder and transferred to the film (left) or biotinylated ladder (right). (E) Total p38 was detected at 37 kDa using the biotinylated ladder. (TIF) [file pone.0125387.s002.tif]
